# A Systematic Review of the Emerging Treatment for Hepatorenal Syndrome With a Principal Focus on Terlipressin: A Recent FDA-Approved Drug

**DOI:** 10.7759/cureus.42367

**Published:** 2023-07-24

**Authors:** Santhosh Raja Thangaraj, Mirra Srinivasan, Hadia Arzoun, Siji S Thomas

**Affiliations:** 1 Internal Medicine, Rajah Muthiah Medical College and Hospital, Chidambaram, IND; 2 Internal Medicine, St. Bernards Medical Center, Jonesboro, USA; 3 Internal Medicine, California Institute of Behavioral Neurosciences & Psychology, Fairfield, USA

**Keywords:** cirrhosis, albumin, norepinephrine, octreotide, midodrine, terlipressin, hepatorenal syndrome

## Abstract

Background: Hepatorenal syndrome (HRS), a consequence of liver cirrhosis, is the development of renal failure, which carries a grave prognosis. Reversing acute renal failure with various vasoconstrictor therapies at an appropriate time favors a good prognosis, especially when a liver transplant is not feasible.

Objective: This study aims to compare various treatment modalities to deduce an effective way to manage HRS.

Methods: The authors conducted a literature search in PubMed, Google Scholar, the Cochrane Library, and Science Direct in October 2022, using regular and MeSH keywords. A total of 1072 articles were identified. The PRISMA guidelines were used, the PICO framework was addressed, and the inclusion criteria were set based on studies from the past 10 years. After quality assessment, 14 studies were included for in-depth analysis in this review.

Results: A total of 14 studies were included after quality assessment, including randomized controlled trials, systematic reviews, meta-analyses, and observational cohort studies. Nine hundred and forty-one patients represented this review's experimental and observational studies, apart from the other systematic reviews analyzed. Nine studies discovered that Terlipressin, especially when administered with albumin, was more effective than other conventional treatment modalities, including norepinephrine and midodrine, in terms of improving mortality and reversing the HRS. Four studies suggested that terlipressin exhibited similar effectiveness but found no significant difference. In contrast, one study found that norepinephrine was superior to terlipressin when particularly considering the adverse effects.

Conclusion: Terlipressin, one of the most widely used vasoconstrictor agents across the world, seems to be effective in reversing renal failure in HRS. Although adverse effects are seen with this agent, it is still beneficial when compared to other medications. Further studies with larger sample sizes may be warranted.

## Introduction and background

**Figure 1:**
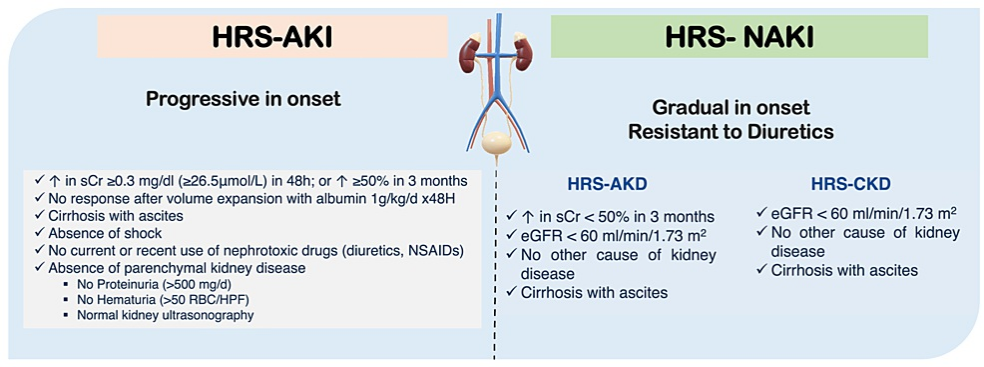
The differences between HRS-AKI vs. HRS-NAKI Image created by the authors using Microsoft PowerPoint HRS: Hepatorenal syndrome; AKI: Acute kidney injury; NAKI: Non-acute kidney injury; sCr: Serum creatinine; AKD: Acute kidney disease; CKD: Chronic kidney disease; eGFR: Estimated glomerular filtration rate

Hepatorenal syndrome (HRS), a form of acute kidney injury (AKI), defined as an increase in serum creatinine of at least 50% from baseline to a value of at least 1.5 mg/dL [1,2], is fatal, with high rates of morbidity and mortality [3]. It is a reversible complication seen in advanced cirrhosis [4]. Frerichs and Flint first described renal function disturbances in liver disease in the 19th century. They identified oliguria in chronic liver disease patients who did not have proteinuria [5,6]. They attributed the renal hypoperfusion to being significantly influenced by the apparent anomalies in endogenous vasoactive systems and systemic arterial circulation [6-9]. Despite reduced renal function, the kidneys' histologic appearance in HRS is normal [7-9]. Figure 1 below depicts more details on HRS-AKI versus HRS-NAKI [10-13].

HRS can also develop in patients with fulminant hepatitis, portal hypertension, and ascites, contributing to hepatic failure in the setting of AKI due to vasoconstriction and hypoperfused kidneys [14-16]. Decompensated cirrhotics have an 8%-20% annual chance of HRS with ascites, which increases to 40% after five years [6]. Vasoconstrictors or liver transplantation could restore adequate blood flow, enhancing renal function [12]. The most notable vasoconstrictor in use today is Terlipressin, a synthetic vasopressin analog used as first-line therapy in several countries [4,17]. It is the first FDA-approved drug for HRS in the United States [18,19]. Others include norepinephrine and midodrine/octreotide [4]. This review analyzes the effectiveness of various treatment options and the benefits vs. risks of Terlipressin in the reversal of HRS.

## Review

Methodology

Data Sources and Searches

A literature search was done on PubMed, Google Scholar, the Cochrane Library, and Science Direct in October 2022. A total of 1072 articles were identified. The following keywords were used.

Regular Keywords

Hepatorenal syndrome, Terlipressin, midodrine, octreotide, norepinephrine, albumin, cirrhosis

MeSH Keywords

Terlipressin OR "Terlipressin/administration and dosage" [Mesh] OR "Terlipressin/adverse effects" [Mesh] OR "Terlipressin/chemistry" [Mesh] OR "Terlipressin/metabolism" [Mesh] OR "Terlipressin/therapeutic use" [Mesh])) AND Hepatorenal Syndrome OR ("Hepatorenal Syndrome/classification" [Mesh] OR "Hepatorenal Syndrome/complications" [Mesh] OR "Hepatorenal Syndrome/diagnosis" [Mesh] OR "Hepatorenal Syndrome/drug therapy" [Mesh] OR "Hepatorenal Syndrome/epidemiology" [Mesh] OR "Hepatorenal Syndrome/etiology" [Mesh] OR "Hepatorenal Syndrome/metabolism" [Mesh] OR "Hepatorenal Syndrome/mortality" [Mesh] OR "Hepatorenal Syndrome/pathology" [Mesh] OR "Hepatorenal Syndrome/physiopathology" [Mesh] OR "Hepatorenal Syndrome/therapy" [Mesh])

Study Selection

The Preferred Reporting Items for Systematic Reviews and Meta-Analysis (PRISMA) guidelines and principles were followed in this systematic review [Page], and the population, intervention, comparison, and outcome (PICO) framework was employed in this study design. The authors included studies from the past 10 years, published worldwide, not limited to English, and explored traditional HRS treatment. Multiple study designs were included, such as observational, experimental, and critical review studies. Articles published before 2012 were not included to maintain the emerging information on managing HRS. Poor-quality studies with overall scores of < 70% were excluded from this study. The population, intervention, comparison, and outcome (PICO) framework was employed, with the population as HRS, the intervention as terlipressin, the control as placebo or other conventional therapy, and the outcome as the reversal of HRS.

Data Extraction and Quality Assessment

Table 1 depicts the type of study reviewed, the respective quality appraisal tools used, and the quality of evidence according to the modified Oxford Center for Evidence-Based Medicine [3,4,7,8,13,20-28].

**Table 1:** Quality appraisal of the included studies RCT: Randomized controlled trial; SR/MA: Systematic review and meta-analysis; MA: Meta-analysis

Author/Year	Type of Study	Quality of Evidence	Quality Appraisal Tool
Pitre et al., 2022 [3]	SR/MA	I	PRISMA Checklist
Koneti et al., 2022 [20]	RCT	I	Cochrane Risk of Bias
Khan et al., 2022 [21]		I	
Jha et al., 2021 [22]		I	
Wong et al., 2021 [23]		I	
Mohamed et al., 2021 [24]	MA	I	PRISMA Checklist
Nguyen-Tat et al., 2019 [25]	Cohort	II	New Castle-Ottowa
Wang et al., 2018 [4]	SR/MA	I	PRISMA Checklist
Israelsen et al., 2017 [8]		I	
Sanyal et al., 2017 [26]		I	
Boyer et al., 2016 [7]	RCT	I	Cochrane Risk of Bias
Goyal et al., 2016 [13]		I	
Cavallin et al., 2015 [27]		I	
Junior et al., 2014 [28]	SR/MA	I	PRISMA Checklist

Table 2 below depicts the Cochrane Risk of Bias 2.0 tool used for the final included RCTs [7,13,20-23,27].

**Table 2:** The Cochrane Risk of Bias 2.0 for randomized controlled trials

Study	Domain 1	Domain 2	Domain 3	Domain 4	Domain 5	Overall
Koneti et al., [20]	✚	?	✚	✚	✚	✚
Khan et al., [21]	✚	-	✚	✚	✚	✚
Jha et al., [22]	-	-	✚	✚	✚	✚
Wong et al., [23]	-	✚	✚	✚	✚	✚
Goyal et al., [13]	-	-	✚	✚	✚	✚
Boyer et al., [7]	-	✚	✚	✚	✚	✚
Cavallin et al., [27]	✚	-	✚	✚	✚	✚
Domain 1: Bias arising from the randomization process, Domain 2: Bias due to deviations from intended intervention, Domain 3: Bias due to missing outcome data, Domain 4: Bias in measurement of the outcome, Domain 5: Bias in selection of the reported result.						
Judgement: ✚ Low - Some concerns ? No information x High						

Data Synthesis and Analysis

**Figure 2:**
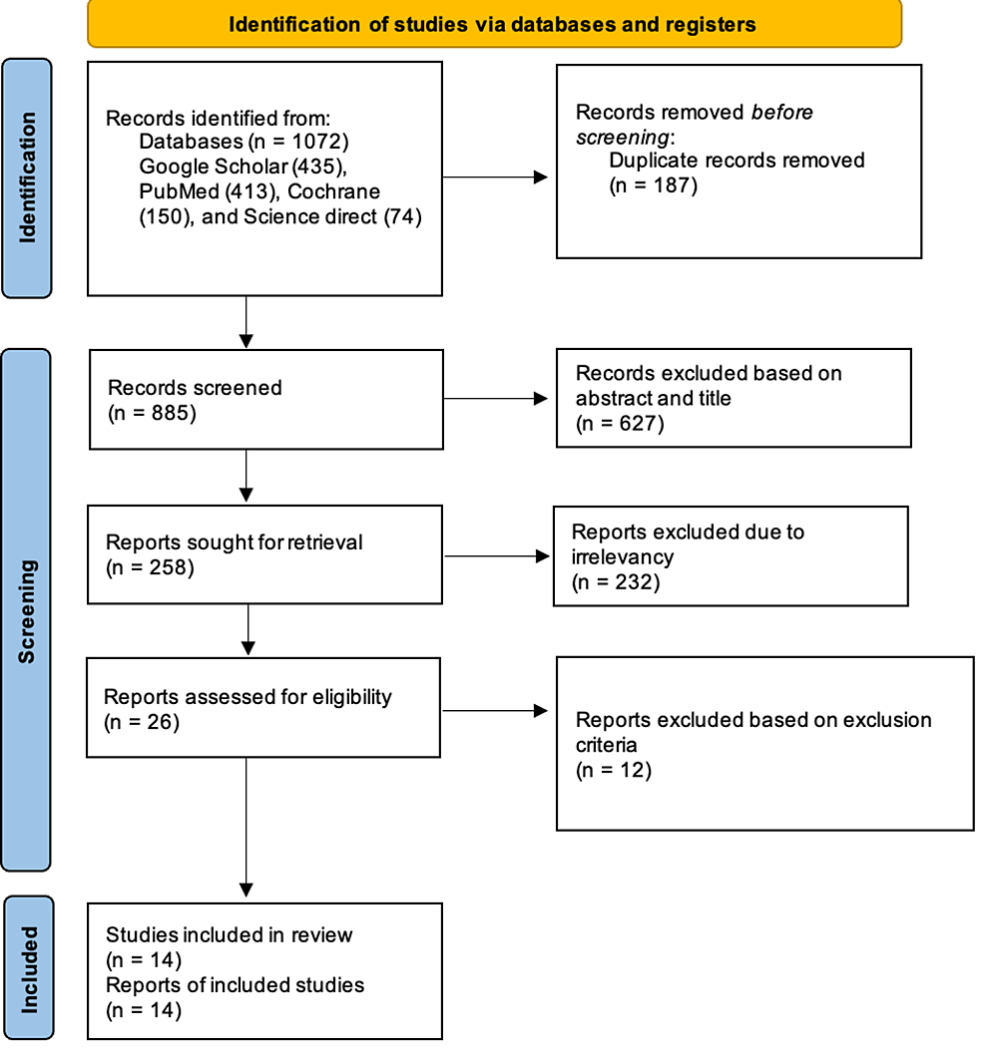
PRISMA flow chart

Among the 1072 articles, 187 duplicates were excluded using EndNote, 885 reports were screened, and 627 were omitted based on the abstract and title, after which 258 reports were sought for retrieval, and 232 reports were omitted due to irrelevancy. The final screening reduced the number of reports to 26, which were evaluated for quality and eligibility. After a thorough reading, 14 eligible reports were included in this study. Two researchers independently extracted and identified data from each study and used the appropriate quality assessment techniques to examine each study's efficacy. When there were differences of opinion, the two researchers considered the study designs, inclusion and exclusion criteria, interventions used, and outcome evaluation to reach a consensus. In ambiguous instances, a third author was brought in to settle disagreements and reach an agreement. A total of 13 reports were eventually included in this study after a thorough investigation. This study did not use any automation tools. Figure 2 depicts the search process used for this review in the form of a PRISMA flow diagram [29].

Results

Table 3 summarizes the results for each study included in this review [3,4,7,8,13,20-28]. 

**Table 3:** Summary of results PEP: Primary endpoint; SEP: Secondary endpoint; SCr: Serum creatinine; HRS: Hepatorenal syndrome; AE: Adverse events; CHRSR: Confirmed hepatorenal syndrome reversal; RRT: Renal replacement therapy; OS: Overall survival; LTx: Liver transplant; MELD: Model for end-stage liver disease; MAP: Mean arterial pressure; RCTs: Randomized controlled trials; SIRS: Systemic inflammatory response syndrome; PRA: Plasma renin activity

Author/Year	Primary End Point (PEP)	Secondary End Point (SEP)	Findings	Conclusion
Pitre et al., 2022 [3]	All-cause mortality HRS reversal Major AE	-	Terlipressin - 142 reversals per 1000 [95% CI, >87.7 to >210.9]; high certainty. Norepinephrine- 112.7 reversals per 1,000 [95% CI, 52.6 to >192.3]; low certainty. Midodrine plus octreotide - 67.8 reversals per 1,000 [95% CI, <2.8 to >177.4]; very low certainty. Terlipressin - 93.7 fewer deaths [95% CI, 168.7 to <12.5]; low certainty. Terlipressin - 20.4 more AE per 1,000 [95% CI, <5.1 to >51]; moderate certainty.	Terlipressin accelerates HRS reversal and could lower mortality.
Koneti et al., 2022 [20]	HRS reversal	AE progression to liver transplantation mortality cost-effectiveness	Norepinephrine: 33 (54.1%), Terlipressin: 28 (45.9%); p = 0.205. Norepinephrine was comparatively cost-effective; p < 0.001. No significant difference in AE, progression to LTx, or mortality. LTx - 29 patients (15 in Norepinephrine and 14 in Terlipressin), with a 3.4% (n=6) 3-month mortality.	When combined with albumin and Midodrine, norepinephrine was as effective as terlipressin.
Khan et al., 2022 [21]	HRS reversal	-	Terlipressin had nearly 78% effectiveness, whereas albumin alone demonstrated 47%. No significant difference in mortality rate was observed in three-month follow-up with a p-value<0.05.	Terlipressin with albumin was shown to be more beneficial than albumin alone.
Jha et al., 2021 [22]	Improvement in SCr	Increase in MAP, urine output; decrease in PRA and aldosterone concentrations	Terlipressin- 8 (40%); Norepinephrine- 10 (50%) responded to therapy. Both groups showed a significant decrease in SCr from baseline, an increase in MAP and urine output, and a substantial reduction in PRA and aldosterone concentrations at day 15.	Norepinephrine and terlipressin were equally effective.
Wong et al., 2021 [23]	HRS reversal	HRS reversal, defined as any SCr ≤ 1.5 mg/dL during the first 14 days; HRS reversal without RRT by day 30; HRS reversal in patients with SIRS; verified HRS reversal without HRS recurrence by day 30.	PEP: Reversal of HRS: Terlipressin = 63/199 (32%), and Placebo 17/100 (17%), p = 0.006. SEP: Terlipressin: 78/199 (39%) vs. Placebo: 18/101(18%); p < 0.001; Terlipressin: 68/199 (34%) vs. Placebo: 17/101 (17%); p = 0.001) Terlipressin: 31/84 (37%) vs. Placebo: 3/48 (6%); p < 0.001) Terlipressin: 52//199 (26%) vs. Placebo: 17/101 (17%); p = 0.08	Terlipressin was more effective but associated with severe AE, including respiratory failure.
Mohammed et al., 2021 [24]	HRS reversal	Change in SCr RRT at 30 days of randomization 90-day survival.	Terlipressin – Better HRS reversal (RR 2.08; 95% CI [1.51, 2.86], p < 0.001), significantly lower serum Cr (mean difference -0.64; 95% CI (1.02,0.27), p < 0.001), fewer RRT requirements (RR 0.61; 95% CI [0.36, 1.02], p = 0.06). At 90 days, no difference in survival between groups (RR 1.09; 95% CI (0.84,1.43), p = 0.52).	Terlipressin was found to be effective in reversing HRS; however, a 90-day survival benefit was not seen.
Nguyen-Tat et al., 2019 [25]	HRS reversal in type 1 vs. type 2	-	Terlipressin reversed 48% in type 1 and 46% in type 2; (p = 0.84); HRS recurrence is 8% in type 1 and 50% in type 2; p= 0.001 Types 1 and 2 had equivalent OS and LTx-free survival (p = 0.69; p = 0.64). Response to therapy was independently related to improved OS in type 2.	Terlipressin was effective in HRS 2, especially when on the transplant list.
Wang et al., 2018 [4]	HRS reversal, renal function change, and mortality	HRS recurrence AE	Terlipressin - 42.0% Non-Terlipressin-26.2% Terlipressin outperformed placebo and octreotide. Terlipressin is as effective as Norepinephrine but has more AE. No discernible difference between Terlipressin and dopamine.	Terlipressin had a higher caliber than placebo, and octreotide, however, was inferior to Norepinephrine.
Israelsen et al., 2017 [8]	All-cause mortality Persistent HRS despite treatment Serious AE	Health-related quality of life Non-serious AE	Terlipressin- no significant difference in mortality. Terlipressin was found to be effective in one meta-analysis of nine studies. Terlipressin appeared to increase the likelihood of diarrhea, abdominal discomfort, or both (RR 3.50, 95% CI 1.19 to 10.27). Subgroup analysis indicated that terlipressin was superior to midodrine and octreotide (RR 0.47, 95% CI 0.30 to 0.72) or octreotide alone (RR 0.56, 95% CI 0.33 to 0.96).	There is insufficient data to support or dispute terlipressin and albumin's positive or negative effects compared to other vasoactive medications.
Sanyal et al., 2017 [26]	HRS reversal	-	Terlipressin – 27%; Placebo 14%; p = 0.004. Terlipressin was associated with a more significant improvement in renal function from baseline until the end of treatment, with a mean between-group difference in SCr concentration of 53.0 µmol/L (P < 0.0001).	Terlipressin seemed to improve renal function better.
Boyer et al., 2016 [7]	CHRSR, defined as two SCr readings ≤1.5 mg/dl spaced 40 hours apart on treatment without RRT or LTx.	HRS reversal (at least one SCr value ≤ 1.5 mg/dL while on therapy). Transplant-free survival; Overall survival	CHRSR: Terlipressin: 19/97 (19.6%), Placebo: 13/99 (13.1%); p = 0.22 SEP: Terlipressin: 23/97 (23.7%), Placebo: 15/99 (15.2%); p = 0.13. SCr decreased by 1.1 mg/dL in Terlipressin, 0.6 mg/dL in placebo-treated patients (p < 0.001). Reduction in SCr and survival were correlated (r (2) = 0.882; p < 0.001). Overall and transplant-free survival was comparable across groups.	Terlipressin + albumin was associated with significantly improved renal function than albumin alone.
Goyal et al., 2016 [13]	HRS reversal	Completion of two weeks of therapy or liver transplantation or death.	Norepinephrine: 47.6% (10/21); Terlipressin 45% (9/20); p = 1.00. In both groups, there was a significant reduction in SCr from baseline (Norepinephrine - 3.1±1.4 mg/dl to 2.2±1.3 mg/dl, p = 0.028; Terlipressin - 3.4±1.6 mg/dl to 2.3±1.3 mg/dl, p = 0.035). Both showed a significant increase in MAP (p=0.0001).	Norepinephrine is as effective and safe as terlipressin.
Cavallin et al., 2015 [27]	Reversal of renal failure	Survival at one and three months following treatment	Significant improvement in renal function was more with terlipressin: 19/27 (70.4%) compared to the midodrine plus albumin: 6/21 (28.6%); p = 0.01. Improved renal function and lower baseline MELD score were associated with better survival.	Terlipressin + albumin is significantly more effective than midodrine.
Junior et al., 2014 [28]	HRS Reversal	Mortality recurrence of HRS AE	No difference between norepinephrine and terlipressin in HRS reversal (RR = 0.97, 95% CI = 0.76 to 1.23), mortality at 30 days (RR = 0.89, 95% CI = 0.68 to 1.17) or HRS recurrence (RR = 0.72, 95% CI = 0.36 to 1.45). With Norepinephrine, AE were less prevalent (RR = 0.36, 95% CI = 0.15 to 0.83).	Norepinephrine is as effective as terlipressin, with fewer AE.

Discussion

The pathophysiology of HRS is not fully known [30]; however, it is considered a multifactorial triggering event [31,32], where the vascular system plays a crucial role. Cirrhosis gradually increases portal venous resistance, which results in increased blood flow in the splanchnic circulation, further releasing vasodilators, including nitric oxide [30,33], in turn reducing the mean arterial pressure (MAP) and circulatory volume [31]. The fluid retention is brought on by increased anti-diuretic hormone and a decrease in glomerular filtration rate [28]. The declining systemic circulation counter-responses by activating the sympathetic nervous system, the renin-angiotensin-aldosterone system (RAAS) (increasing the circulating angiotensin II levels), and the release of arginine vasopressin, but at the expense of severe constriction of the renal vasculature, which results in a progressive fulminant form of AKI [30,31,34-36].

**Figure 3:**
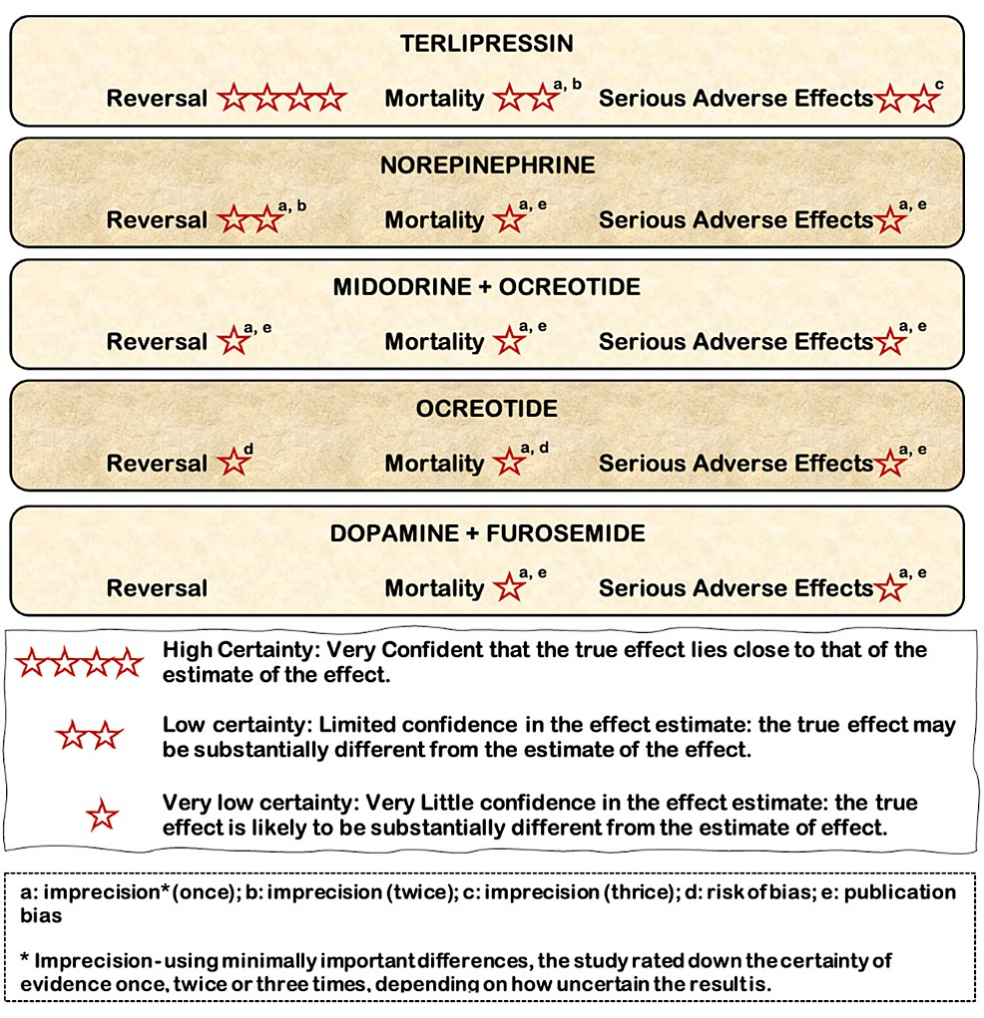
Depicts various RCTs' drug nodes versus placebo Original image was created by the authors using Microsoft PowerPoint.

A liver transplant, the only effective treatment, resolves severe liver disease and portal hypertension, leading to renal recovery. However, only a tiny percentage of HRS patients qualify for timely LTx [31]. HRS can also be treated with various other modalities, including vasoconstrictors, albumin, transjugular intrahepatic portosystemic stent-shunt (TIPSS) [37], and extracorporeal albumin dialysis; however, vasoconstrictors are the most popular approach due to their efficacy and practicality [4,38]. Renal function can be improved by splanchnic vasoconstrictors and albumin therapy, which may also increase short-term waitlist survival [31,39]. Terlipressin causes splanchnic vasoconstriction, which diverts blood to the systemic circulation, lowers the sympathetic nervous system and RAAS activation, decreases the production of arginine vasopressin, and eventually improves kidney perfusion [30,38,40-43]. Norepinephrine causes vasoconstriction with minimal effects on the myocardium and corrects the low systemic vascular resistance associated with HRS [41]. Midodrine causes systemic vasoconstriction that, in turn, improves systemic blood pressure and enhances renal perfusion pressure. Octreotide counteracts the effects of several splanchnic vasodilators and decreases the discrepancy in intravascular volume and arterial vasodilation [30,38,40-43]. Figure 3 below illustrates the drug nodes versus placebo, presented as high, moderate, low, or very low certainty, with data incorporated from 26 RCTs [3].

In the absence of terlipressin, norepinephrine use for HRS may be more favorable compared to the conventional combination of octreotide and midodrine. However, norepinephrine frequently necessitates admission to an intensive care or high-dependency facility, which is linked with increased expenditures and resources; hence, octreotide and midodrine are commonly utilized as less expensive alternatives [44]. The persistence of HRS reversal with terlipressin remained until day 30 without renal-replacement therapy (RRT) [42]; this seems therapeutically noteworthy since RRT poses several difficulties for individuals with advanced cirrhosis. It has been hypothesized that terlipressin, by its vasoconstrictor effect, decreases portal inflow [45], protects against bacterial translocation, endotoxemia, and ensuing pro-inflammatory cytokines, which most likely enhances terlipressin's response in patients with decompensated liver cirrhosis [24,42].

The inclusion of the recently released CONFIRM study showed terlipressin to be very beneficial in reversing HRS [3,42]. Compared to the placebo group, the incidence of major adverse events was more significant in the terlipressin group. Potential side effects of terlipressin include abdominal pain, skin discoloration, intestinal ischemia, cardiac ischemia, cyanosis, bradycardia, and diarrhea. The terlipressin group demonstrated increased gastrointestinal bleeding (4% vs. 0%), sepsis (4% vs. 0%), and respiratory failure (10% vs. 3%) compared to the placebo group [42]. Terlipressin's known cardiovascular and pulmonary effects may be responsible for the higher rates of respiratory failure in the terlipressin group compared to the placebo group [42]. Before CONFIRM, most of the terlipressin data came from smaller, nonblinded RCTs with low event rates and unreliable impact estimates [3].

Junior et al. state that in the included trials, only two of the nine cardiovascular events (episodes of segment ST depression) resulted in a change in medication (a titration of dose). The frequencies of adverse events were lower for norepinephrine than terlipressin, as reported in previous studies [4,28,46]. The higher incidence of adverse effects observed in the terlipressin group compared to the norepinephrine group could be explained by three factors. First, the terlipressin dosage was high in studies including people with HRS-2 [47], which may lead to more side effects. Second, the in-depth analysis revealed that these RCTs needed more data on adverse events. Third, terlipressin's side effects were inevitably added to the RCTs with documented adverse events because placebo and norepinephrine hardly had any specificity complications [48,49]. Most studies only mentioned terlipressin-specific issues, like abdominal cramps and arrhythmia [4]. Terlipressin can be administered as an intravenous bolus peripherally and hence may be safely administered in regular wards without risk, although it is expensive. But norepinephrine is often administered intravenously as a continuous infusion through a central venous catheter, which requires intensive care unit-level care [28].

The superior effectiveness of terlipressin plus albumin over midodrine and octreotide (MID/OCT) plus albumin in improving renal function may be explained by the more significant effect of terlipressin treatment on increasing MAP [27]. The fact that there were no variations in adverse events between the groups is substantial. Some might counter that in the trial by Sanyal et al., the rate of complete response in the MID/OCT group patients was even lower than that seen with albumin alone [26].

In one study, 50% of all treated patients experienced a recurrence of HRS, while other studies found that relapse rates were between 35% and >50% [25]. The higher recurrence rate after vasoconstrictor withdrawal most likely reflects the fact that HRS type 2 develops in a state of persistent portal hypertension in patients with refractory ascites, as opposed to HRS type 1, which typically develops after an acute complication like infection or GI bleeding with a potentially reversible decompensation of liver and renal function. Relapse patients who underwent retreatment have seen a 43% response rate, corroborating the theory that terlipressin therapy can be used as a stopgap measure before transplant in LTx-eligible patients [25].

Several vasoconstrictors have demonstrated favorable outcomes in the treatment of type 1 HRS; however, there have been relatively few trials on the use of vasopressors in treating type 2 HRS. Terlipressin has been officially approved as one of the mainstay medications to treat HRS. The United States Food and Drug Administration (FDA) recently approved terlipressin injections in adults with HRS with a rapid reduction in kidney function. Terlipressin, to date, remains the first FDA-approved medication for this condition [18]. The risk of severe or fatal respiratory failure increases with terlipressin. Patients with low blood oxygen levels shouldn't be started on this drug [50]. Using a pulse oximeter while receiving this therapy is essential, and clinicians must remain vigilant for patients for breathing issues [1,18,50]. 

Limitations

Due to the inability to assess the complete text, few studies were excluded; however, the comparison between different treatment options was analyzed. Since the authors included a worldwide search, the method of practicing and diagnosing HRS could have been different across hospitals worldwide. The initiation of treatment could not be studied in depth due to this limitation. Other limitations include the lack of a control group in the observation study, the RCT's small sample size, and minimal information on adverse effects. The mortality estimate is likely confounded by eligibility and the receipt of a liver transplant.

## Conclusions

HRS ultimately leads to fluid overload, secondary infection, and organ damage, which may even be fatal without treatment. When a liver transplant is not feasible, medical management is the ultimate resort. Terlipressin seems to be effective in reversing renal failure in HRS and may even decrease mortality. Although adverse effects are seen with this agent, it still seems to be beneficial when compared to other conventional medications. However, further studies with larger sample sizes may be warranted, especially since the adverse events are to be explored in depth.
